# Loeys-Dietz Syndrome Presenting as Hemorrhage From Arteriobiliary Fistula: Endoscopic Management of Hemostasis

**DOI:** 10.1016/j.gastha.2025.100794

**Published:** 2025-09-05

**Authors:** Yilin Song, Farhan Ali, Eric M. Goldberg

**Affiliations:** 1 Department of Internal Medicine, University of Maryland Medical Center Midtown Campus, Baltimore, Maryland; 2 Department of Endocrinology Research Unit, Mayo Clinic, Rochester, Minnesota; 3 Department of Gastroenterology and Hepatology, University of Maryland Medical Center, Baltimore, Maryland

**Keywords:** Arteriobiliary Fistula, Loeys-Dietz Syndrome, Metal-Covered Stent, Refractory Hemorrhage

## Abstract

Loeys-Dietz syndrome is a rare autosomal dominant connective tissue disorder with an increasing risk of vascular aneurysm formation and dissection. In this report, we discuss the novel therapeutic use of a fully covered metallic stent for management of severe refractory hemorrhage from arteriobiliary fistula in a case complicated with Loeys-Dietz Syndrome and post thoracic aneurysm repair. Recent history of pancreaticoduodenectomy added on the anatomy complexity, and embolization of hepatic artery branches proved unsuccessful. Hemostasis was achieved after a metallic stent placement in the common bile and hepatic duct via endoscopic retrograde cholangiopancreatography.

## Introduction

Loeys-Dietz syndrome (LDS) is a rare autosomal dominant connective tissue disorder first described in 2005.^1^ Vascular aneurysm formation and dissection are common features of LDS.^2^ Among the 5 identified subtypes, type 1 is the most severe involving transforming growth factor β receptor 1 mutation and rapid progression of thoracic aortic aneurysms.^2^ Common hemorrhage sites post pancreaticoduodenectomy (Whipple procedure) include blood vessels, anastomotic sites, pancreatic stump surface, and retroperitoneum.^3^, ^4^, ^5^, ^6^ Arteriobiliary fistula was identified as the hemorrhage source in the current case and can result from vascular anomalies such as hepatic artery pseudoaneurysms.^7^ There was no report of LDS leading to the formation of arteriobiliary fistula. However, given the vascular fragility, arteriobiliary fistula formation is plausible in the setting of LDS.

Hemorrhage can be managed conservatively or via procedural intervention.^4^ Embolization of hepatic artery branches with glue and coil proved unsuccessful before metallic stent placement across the hepatic duct via endoscopic retrograde cholangiopancreatography (ERCP). Our case highlights the complexity of localizing and managing hemorrhage in patients with LDS post-Whipple procedure, which may lead to delayed diagnosis, treatment and increased mortality.

## Case Report

A 35-year-old male presented with hematemesis, abdominal and upper back pain. He was transferred due to refractory gastroenterological bleeding. Past medical history was remarkable for hypertension, pancreatic neuroendocrine tumor post-Whipple procedure (8 months prior), type B aortic aneurysm post thoracic endovascular aortic repair and carotid stenosis post carotid-subclavian bypass (7 months prior).

**Figure 1:**
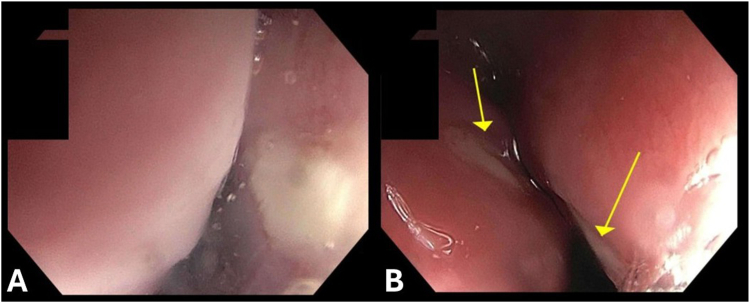
(A, B) Two esophageal kissing ulcers without signs of active bleeding.

The patient had an episode of hematochezia complicated by pulseless cardiac arrest 2 months prior to admission. Esophagogastroduodenoscopy (EGD) at that time revealed active bleeding from the biliopancreatic limb of gastrojejunostomy. Hemoglobin dropped to 4.9 g/dL requiring multiple red blood cell transfusions. The patient had repeated episodes of hematochezia and hematemesis, and visceral angiography revealed an arteriobiliary fistula. Glue and coil embolization of hepatic artery branches was performed. He remained slightly above the transfusion threshold for 48 h before having another episode of hematemesis. EGD identified nonbleeding esophageal ulcers (Figure 1) but no identifiable source of active bleeding. Subsequently, the patient had an episode of hematochezia with negative findings on computed tomography angiography. Tagged nuclear medicine red blood cell scan showed active bleeding into the small bowel. With refractory bleeding and decreasing hemoglobin (6.1 g/dL), the patient was transferred to University of Maryland Medical Center.

**Figure 2:**
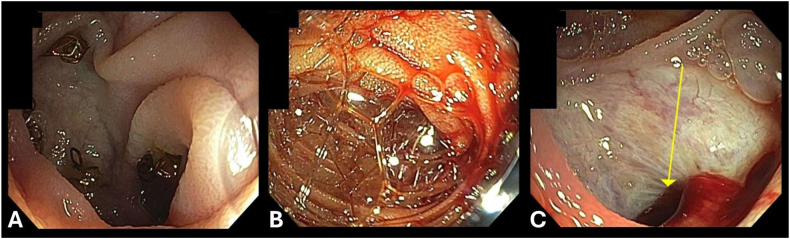
(A) Staples from previous Whipple procedure at afferent limb of gastrojejunal anastomosis. (B) Fresh blood seen at the afferent limb. (C) Active bleeding and hemobilia were observed at the opening of the biliary to enteric anastomosis site.

**Figure 3:**
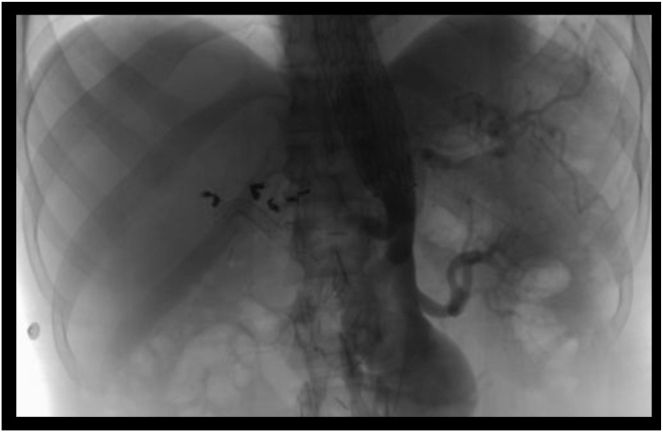
Beaded appearance of mesenteric vasculature, compatible with large and medium vessel vasculitis.

**Figure 4:**
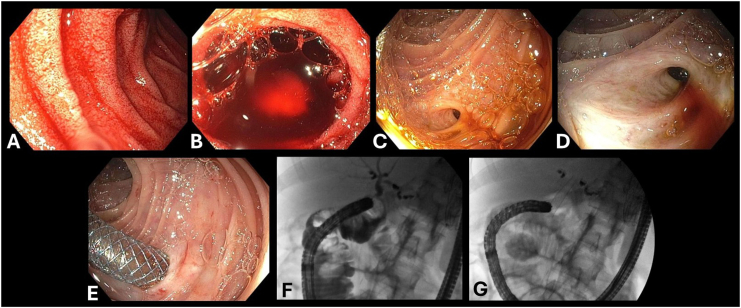
(A, B) Pool of blood showed in afferent limb of gastrojejunal anastomosis. (C, D) Active bleeding and bile from orifice at choledochojejunostomy. (E) 10 mm by 4 cm covered metal stent was placed 2.5 cm into the common bile duct, bleeding controlled via tamponade. (F) Common hepatic duct shown with contrast. (G) Metal-covered stent placement.

Endoscopy showed active bleeding from afferent limb. Fresh blood emanating from the bilioenteric anastomosis orifice consistent with hemobilia, confirming the presence of arteriobiliary fistula (Figure 2). Angiogram showed diffuse beading of arterial vasculature considering vasculitis (Figure 3). LDS was diagnosed with positive transforming growth factor β receptor 1 mutation. ERCP was performed and active bleeding was observed from the hepatic-jejunostomy in the post-Whipple afferent limb. One 10 mm by 4 cm fully covered metal stent was placed 2.5 cm into the common bile duct to tamponade the bleeding source (Figure 4). The patient was stabilized and discharged 4 days after the procedure. A repeated ERCP was performed 2 months after with stent removal. No recurrent bleeding episode occurred after the initial ERCP and stent removal.

The patient’s clinical course comprised 76 days during which he required 25 units of blood products. Hemostasis was eventually achieved after stent placement. The patient expressed thankfulness for the treatment and gave consent to the case report.

## Discussion

LDS is an autosomal dominant connective tissue disorder reported in 2005.^1^ Five subtypes have been identified.^2^ Rapid progression of aortic aneurysm is a distinct feature of LDS, which can be further complicated by aneurysm dissection in 17%–25% of cases.^2^^,^^8^ The prevalence of dissection is specifically high among thoracic aortic aneurysms.^9^ Our case was complicated with type 1 LDS and vasculitis, thought to be the main contributing factor to the refractory hemorrhage.

There was no previous report on LDS complicated with vasculitis. Vascular disease is a common feature of LDS, especially thoracic aortic aneurysms.^2^ Vasculitis diseases such as Kawasaki and Takayasu disease can cause artery aneurysms. Uncommon presentation with advanced severity or early onset age should arise concern of LDS.^8^ Similarly, no previous report of LDS leading to the formation of arteriobiliary fistula. A case report identified arteriobiliary fistula resulting from hepatic artery pseudoaneurysms in a patient without LDS.^7^ With negative vasculitis antibody workup, our hypothesis is that vascular fragility and aneurysm formation in the setting of LDS leading to arteriobiliary fistula formation.

Whipple procedure is another possible contributing factor to the current case, which carries multiple postsurgical complications. Hemorrhage is less common but carries a mortality rate of 12.5%–60%.^3^^,^^4^^,^^6^ Postsurgical hemorrhage can be delayed up to 12 weeks.^6^^,^^10^^,^^11^ Severe bleeding requires surgical treatment and can lead to cardiac arrest.^5^ The hemorrhage in the current case occurred 8-month post-Whipple procedure, which was challenging to control due to the anatomy complexity.

The reported hemorrhage locations include arteries, veins, anastomotic sites, pancreatic cut surface, and retroperitoneum.^3^, ^4^, ^5^, ^6^ Angiography or EGD are utilized to locate the hemorrhage site, but not all locations can be identified.^4^^,^^11^^,^^12^ Arteriobiliary fistula and hemobilia are rare upper gastrointestinal tract bleeding sources which were reported as complications of transjugular intrahepatic portosystemic shunt, chemoradiation therapy of intrahepatic cholangiocarcinoma or hepatic artery pseudoaneurysm after liver transplantation.^7^^,^^13^^,^^14^

Management methods include medication, arterial embolization, endoscopy, and surgery.^12^ Embolization with coils or glue can be applied if a single feeding vessel is identified and with sacrificable local blood flow.^15^ Due to the complexity of post-Whipple procedure anatomy, cases should be approached in a multidisciplinary fashion. Interventional radiology, transplant hepatology, surgical oncology and vascular surgery were consulted before proceeding with endoscopic intervention.

Endoscopic interventions include balloon or chemical tamponade, electrocoagulation, and hemoclip application.^16^ The advantages of stent therapy are preservation of distal blood supply, mechanical tamponade and relief of biliary obstruction.^17^^,^^18^ The rationale for utilizing fully covered metal stents is based on the effectiveness in controlling postsphincterotomy bleeding.^16^

Delayed hemorrhage is a potentially fatal complication of vasculopathy caused by LDS in the setting of anatomy complexity post-Whipple procedure. Our case highlights the challenges associated with both locating the source of bleeding and utilizing various methods to control active hemorrhage.
